# Post-Mortem Cæsarean Section

**Published:** 1916-07-22

**Authors:** 


### POST-MORTEM CÆSAREAN SECTION.

### A Question of Law for Institutional Officers.

It says a great deal for the tact and discretion of institutional medical officers, and particularly for residents (who are usually young and of comparatively brief experience) that actions at law for professional malpraxis against hospitals are very rare, and that on many points there are absolutely no leading cases to guide the authors of medico-legal text-books. A curious and interesting instance of the doubt which surrounds the legal aspects of certain uncommon but not by any means unimaginable contingencies is furnished by post-mortem Cæsarean section, which has been the subject of an illuminating discussion at the New York Academy of Medicine.* The opinions there put forward, both by medical men and by lawyers, do not bind the American courts; and *a fortiori* are no guarantee of the view a British court would take. But the dispassionate consideration beforehand of the various aspects of this subject, and the formulation of rules for conduct by eminent and responsible authorities, might materially influence the views of a judge and jury called upon to investigate the conduct of a medical man who had had to face one or other of these emergencies; and post-mortem Cæsarean section is distinctly a matter upon which resident medical officers of institutions should have clearly defined guidance, rare though the occasion for it unquestionably must be.

### The Indications for the Operation.

Post-mortem Cæsarean section consists in the delivery of an unborn child through an abdominal incision after the death of the mother. To have even an outside chance of saving the infant's life, this operation should be performed within twenty minutes after death, and preferably much sooner than that. The cause of death must have been sudden, and of a nature not in itself liable to injure the fœtus. Thus, sudden death from apoplexy, rapidly fatal injury caused by external violence, embolism, and sudden cardiac dilatation from old valvular disease are favourable cases for its performance; whereas deaths from enteric fever, peritonitis, diabetes, pneumonia, are examples of the opposite; and from placenta prævia, accidental hæmorrhage, rupture of the uterus are intermediate between the two. The nearer the gestation has approached to full term, the better chance is there for a successful issue of the operation.

The necessity or opportunity of performing Cæsarean section is not frequent, because women shortly about to become mothers seldom fall victims to those sudden catastrophes in which the operation is indicated; 123 cases, however, have been collected from the literature in a period of about fifty years (1862-1914) by two authors. Ten cases are reported by Harrar* from one single institution, the New York Lying-in Hospital, out of a total of 91,000 deliveries; and in view of these latter statistics it is probable that many opportunities are missed for lack of initiative, or else that many of these operations go unreported. Hallman in 1914 found the percentage of living children obtained to be between 60 and 70 per cent.; probably this estimate is too high, since failures are more likely to go unreported than successes. In the ten New York cases, three infants were born dead; four were born with hearts beating, but could not be resuscitated; one lived six days; and the remaining two did well. The statistics at least show that the legality of post-mortem Cæsarean section, and the circumstances in which a successful result is possible, are worth the serious attention of the resident medical officers of lying-in institutions.

### The Responsibility of the Surgeon.

To begin with, the doctor confronted with a case in which this operation has a chance of success should, if possible, secure the consent of the deceased woman's husband or family. But if this is not to be had, or is refused, it is not only permissible but obligatory for the operation to be performed without it; so, at least, say the American authorities. Indeed, Mr. G. W. Whiteside, a well-known New York lawyer, carries out his analysis to its logical conclusion that the omission to perform post-mortem Cæsarean section when there is a chance of saving the child's life constitutes at least "manslaughter of the second degree." This view of the surgeon's responsibility he admits to be purely speculative, but his arguments in support of it are based upon first principles and are distinctly attractive. "Inasmuch," says Mr. Whiteside, "as our statutes are interpreted in the light of scientific progress, what might not have been considered as culpable negligence half a century ago, to-day would be considered as gross culpable negligence. This principle is well founded in precedent, and has found expression in cases where safety devices of recent invention . . . are omitted from use by those upon whom responsibility for their use rests. . . . This is particularly true in the engineering field, and its application to medical science but awaits the opportunity which a specific case may afford."

### Legal Complications of Inheritance.

So much for the criminal law of the matter. The civil complications are more numerous. One of them is, Does a child thus delivered after its mother's death share in her estate if she be intestate or if her will provides for a distribution among her children living at the time of her death? It is thought that in United States law such a child would share, though there is as yet no leading case; one of the largest New York life insurance companies has declared that it would act upon

* Reported in the *American Journal of Obstetrics and Gynæcology*, June 1916; pp. 1046-1058 and 1126-1127.

that view without question. We need not go into the arguments with which this assumption is supported, the more so in that they hinge more on the exact wording of certain statutes of the United States than on the basic principles of jurisprudence

Another point in the law of inheritance might arise when an estate is devised to a person for life, and in the event of that person's death without issue then to another person. Suppose the tenant for life be a woman about to bear her first child, which in consequence of her death is delivered by post-mortem Cæsarean section; does the child succeed to the estate? Again the American lawyers say Yes, an answer which at least accords with common sense and fairness. In the same way it is suggested that if a child, born after the death of its mother through the negligence of another, is the woman's only next-of-kin (the husband being presumed dead already), an action can be instituted on its behalf for damages through the death of the mother. If there be no executor an administrator can be appointed for the purpose of upholding the rights of the infant. Once more pushing the argument to the uttermost, such a child might recover damages from a surgeon through whose negligence the death of its mother could be proved to have been brought about.

Whenever circumstances arise which call for this operation, prompt decision and immediate action are needed. To spend precious minutes trying to hear the fœtal heart is to lose the chance of success. No aseptic precautions are necessary, since the patient is already dead: with any cutting instrument that is handy the abdomen and uterus should be freely and quickly incised and the fœtus extracted. The second point to be emphasised is the importance of artificial respiration, kept up as long as any cardiac contraction can be felt by pressing the fingers up under the child's left costal margin. The children are almost always born in severe asphyxia, and great patience is required in their resuscitation. Dr. Harrar states that women in a cataleptic state have been submitted to the operation when believed by the operator to be dead. The first incision, of course, proved that the contrary was the case; the subsequent legal complications of such a *contretemps* were not considered at the meeting in New York, though almost every other possible aspect and incident connected with the question was investigated.

